# Commentary: From Mental Health to Mental Wealth in Athletes: Looking Back and Moving Forward

**DOI:** 10.3389/fpsyg.2017.00693

**Published:** 2017-05-10

**Authors:** Ian Sherwin

**Affiliations:** Department of Physical Education and Sport Sciences, University of LimerickLimerick, Ireland

**Keywords:** mental health, stigma, elite sport, intrinsic motivation, coach education

The mini-review by Uphill et al. highlights the prominence of psychological issues in sport through various media. The review also highlights gaps in the research in conceptions in mental health, interventions needed, and collaborative work that remains outstanding. The review used the Keyes (2002) model as a framework in which athlete well-being and mental health can be readily understood. Although the incidence of mental health difficulties are similar in athletes to the general population the article also draws reference to the existing stigma of mental health and the under-recognition of mental illness in sporting populations. This may be as a result of not wishing to be seen as showing weakness in a culture that perceives athletes as being mentally “tough.” This article focuses on athletes; however, it is important to look beyond athletes as we should be at least as concerned with coaches' well-being as athletes.'

A coach ill-equipped to deal with their own psychological issues may see a change in their coaching behavior which may impact their performance in training and competitive situations. A potential consequence of this would be an increase in negative coaching behaviors (e.g., feedback highlighting athlete mistakes) which may impact their athletes (Badami et al., 2011). High levels of intrinsic motivation are linked to enhanced mental health (Ryan and Deci, 2000). It is known from previous research that coaches who display positive, supportive behaviors increase intrinsic motivation in their athletes (Amorose and Horn, 2000). Conversely, athletes who perceived their coach to display more autocratic behaviors produced lower levels of intrinsic motivation (Hollembeak and Amorose, 2005). Further, the club director-coach (employer–employee) relationship may carry similar interactions that may affect intrinsic motivation in coaches. Previous research in human resources (Boxall et al., 2015) indicates employees who perceive their expectations have been met by employers have a higher level of job satisfaction and are more intrinsically motivated.

While there is advocacy for mental health support services for athletes, evidence of similar services for coaches is scarce. Sebbens et al. (2016) developed a 4-h applied workshop designed to upskill and educate people who work in high performance sport on Mental Health for coaches in Australia which has shown a positive effect on coaches' knowledge of psychological well-being. Even a brief intervention can be effective in increasing coaches' mental health literacy and confidence in helping an athlete in an elite performance setting. Coach–athlete relationships have been deemed essential to effective coaching (Felton and Jowett, 2013). Coaches with a mental health issue (for example, stress) would be predicted to react differently to players' issues at that time which may influence the coach–athlete relationship. For example, should an athlete suffer a career-threatening injury the coach may not have the psychological resources to provide social support to the player if their own well-being is under threat. In contrast, coaches who appraise stress as a challenge are more likely to offer social support to their athletes (Dixon et al., 2016). Similarly there may be unforeseeable factors such as a bereavement that may have a significant impact on the mental health of the entire squad or group. In November 2016, a tragic air accident in South America left few survivors but among them were players and management from the Brazilian Chapecoense soccer team who lost friends, colleagues, and relatives in the ill-fated flight, LMI2933. Where would an incident such as this leave the coaches and players on the spectrum of mental health and well-being? These situations underline the importance of including mental health training in coach education courses so that coaches can have an understanding not just of psychological skills but also of mental health literacy (Sebbens et al., 2016) for themselves, athletes, and others involved in sport.

Coaches are typically hired to achieve an improvement on past team performance (Pierce et al., 2017), rather than to advance the well-being of their players. Thus, it is not surprising that “dark sides” or Machiavellian characteristics may be prominent among successful leaders and coaches (Judge et al., 2009). A coach who displays Machiavellian traits may be more likely to be positioned in the upper left quadrant of the Keyes (2002) model and with support may shift to the right as these traits diminish in favor of a more autonomy-supportive coaching climate. Similarly a coach who is supportive and empathic with athletes and positioned in the upper right quadrant could experience external factors that affect their mental health and fluctuate across the continuum. Coaches who are empathic toward their athletes create an environment of open communication and communicate more effectively (MacIntyre, 2015).

Creating a positive coaching climate and a positive organizational culture (Fletcher and Wagstaff, 2009) must include prioritizing coaches' well-being. Uphill et al.'s (2016) synthesis highlights the upsurge in research on mental health in elite sport (Bauman, 2016; Gucciardi et al., 2016; Rice et al., 2016; Coyle et al., 2017). A gap remains in the research literature which has yet to systematically address mental health in coaches and coaches' understanding of mental health issues in their athletes. Previous research on athlete pathways has shown the need for resilience in athletes as the pathway to performance level is never smooth (Collins et al., 2016). Coaches must have an understanding of the issues that may affect an athlete who perhaps has been deselected from a squad, not selected to start or is struggling with performance. Consequently there is a need for coach educators to address these issues as a priority.

There is an opportunity to present a short intervention such as that alluded to in Sebbens et al. (2016) in all coach education courses which will address both the well-being of coaches and equip them with a basic understanding of athlete mental health issues. Despite a number of high profile tragedies in sport there has been a tendency to downplay the significance of mental health issues (Reardon and Factor, 2010). Support for coaches should be in place not just to address mental health issues but to promote positive mental health within an environment in which a coach can flourish.

## Author contributions

The author confirms being the sole contributor of this work and approved it for publication.

### Conflict of interest statement

The author declares that the research was conducted in the absence of any commercial or financial relationships that could be construed as a potential conflict of interest. The reviewer BO and handling Editor declared their shared affiliation, and the handling Editor states that the process nevertheless met the standards of a fair and objective review.
